# Towards a culture of open scholarship: the role of pedagogical communities

**DOI:** 10.1186/s13104-022-05944-1

**Published:** 2022-02-22

**Authors:** Flávio Azevedo, Meng Liu, Charlotte R. Pennington, Madeleine Pownall, Thomas Rhys Evans, Sam Parsons, Mahmoud Medhat Elsherif, Leticia Micheli, Samuel J. Westwood, Framework for Open, Reproducible Research Training (FORRT)

**Affiliations:** 1 Institute of Communication Science, Friedrich Schiller University, Jena, Germany; 2 Faculty of Education, University of Cambridge, Cambridge, UK; 3 School of Psychology, Aston University, Birmingham, UK; 4 University of Leeds, Woodhouse, UK; 5 University of Greenwich, London, UK; 6 University of Oxford, Oxford, UK; 7 School of Psychology, University of Birmingham, Birmingham, UK; 8 Institute of Psychology, Julius-Maximilians-University of Würzburg, Würzburg, Germany; 9 Department of Psychology, School of Social Science, University of Westminster, London, UK

**Keywords:** Open scholarship, Open educational resources, Open science, Open research, Pedagogy, Reproducibility, Research integrity

## Abstract

The UK House of Commons Science and Technology Committee has called for evidence on the roles that different stakeholders play in reproducibility and research integrity. Of central priority are proposals for improving research integrity and quality, as well as guidance and support for researchers. In response to this, we argue that there is one important component of research integrity that is often absent from discussion: the pedagogical consequences of how we teach, mentor, and supervise students through open scholarship. We justify the need to integrate open scholarship principles into research training within higher education and argue that pedagogical communities play a key role in fostering an inclusive culture of open scholarship. We illustrate these benefits by presenting the *Framework for Open and Reproducible Research Training (FORRT)*, an international grassroots community whose goal is to provide support, resources, visibility, and advocacy for the adoption of principled, open teaching and mentoring practices, whilst generating conversations about the ethics and social impact of higher-education pedagogy. Representing a diverse group of early-career researchers and students across specialisms, we advocate for greater recognition of and support for pedagogical communities, and encourage all research stakeholders to engage with these communities to enable long-term, sustainable change.

## Introduction

The *open scholarship* movement seeks to make knowledge of all kinds openly shared, transparent, rigorously researched, and inclusive [1, 2]. The movement is composed of many grassroots and top-down initiatives that have successfully accelerated adoption of open scholarship practices (e.g., study preregistration, data sharing, replication studies, and open access publishing), bringing well-needed change to research practice. However, wider adoption across disciplines and career stages remains limited, while social injustices in research culture remain a persistent and largely ignored issue [3]. One main reason is that most initiatives only encourage open scholarship and higher standards for quality of evidence [4, 5], but fail to address how we teach, mentor, and supervise students through open scholarship in higher education. By overlooking the opportunity to reshape the future generation of researchers and consumers of science, we undermine the goal towards permanently redressing perverse academic incentives and research evaluations that undermine research quality and an inclusive research culture [6–8].

The above situation is likely to remain unchanged if initiatives that seek to incorporate open scholarly practices in teaching and mentoring continue to receive no support from stakeholders and no recognition or reward from institutional policies and procedures. In a typical University, for example, the time of faculty members and researchers is spread across teaching, research, and administration, and for those on research contracts, is focused on academic outputs, grants and external engagement. This lack of support and reward works as a disincentive to teaching through open scholarship and, therefore, to promoting best practice in research integrity and culture. As a result, even though the wave of scientific reform is influencing scientific practices and norms globally, the current model of higher education is largely outdated with respect to open scholarship with many students finishing their degree without ever learning about the ‘credibility crisis’ or open scholarship practices [9].

We propose that pedagogical communities play a fundamental role in incorporating open scholarship in higher education with the view to improve future research practice and culture. Pedagogical communities are educationally-oriented ‘open science communities’ [10] that make open science knowledge accessible and facilitate communication between academia and policy. They also advocate for the integration of open scholarship into higher education and raise awareness of its pedagogical implications and associated challenges. Pedagogical communities equip educators with the necessary didactic tools to incorporate open scholarship into curricula and educators’ teaching, mentoring, and research practices.

In what follows, we outline the advantages of integrating open scholarship into higher education. We discuss what pedagogical communities can bring to the open scholarship movement, and exemplify their potential benefits with one such community. We call for greater collaboration between pedagogical communities and all the stakeholders of research to minimise the demands of introducing open scholarship pedagogy and to improve—and make future-proof—research integrity.

### What are the benefits of integrating open scholarship into higher education?

Teaching open scholarship benefits students, researchers, and society.

First, undergraduate and postgraduate students in social and health sciences are unnecessarily disadvantaged if they wish to have a research career inside or outside academia. Open scholarship is generally not taught in higher education but is increasingly being practiced in research, and this misalignment is compounded by the fact that the standard practices being taught to students tend to not prioritise research transparency or quality (e.g., by reporting post-hoc analyses as confirmatory, discouraging replication studies, focusing on novel research). By supporting the teaching of open scholarship at the undergraduate and postgraduate level, pedagogical communities help improve the quality of research produced by future generations of career researchers.

Second, from the perspective of researchers, the integration of open and reproducible practices into teaching facilitates the alignment between research belief and research practice. We argue that open research is incomplete without open educational practices. Core values such as openness, transparency, inclusivity, accessibility, and reproducibility are not exclusive to research alone and should be embedded in teaching. Training our future researchers and consumers of science through open scholarship allows open science practices to become the norm and to be passed on to the next generation, cumulatively consolidating the foundation for a more reproducible and inclusive science.

Third, integrating open scholarship into higher education advances social justice which, whilst being the most fundamental, is arguably one of the most overlooked tenets of contemporary scholarship [11]. Indeed, open scholarship, including open educational resources, is underpinned by the powerful idea that knowledge is a public good for all of humanity [11–13]. Current academic systems perpetuate global inequalities with prescribed dogmas, reinforced hierarchies, and hidden curricula. There are still systematic barriers to accessing scientific knowledge, where barriers exist not only between and within institutions but also between academia and the public. Integrating open educational resources into higher education can remove barriers to entry and facilitate career progression by offering students and aspiring scholars accessible and ethically-curated tools to critically engage with the process of science-making, ultimately enhancing diversity and representation within science.

While there are few notable exceptions, e.g., [14–16], attempts to incorporate open scholarship in higher education often require a crowd-sourced, community-based effort. Pedagogical communities exemplify a promising pathway towards a culture of open scholarship practices in research, education and training, empowering individual members of the research community. This includes not only those who conduct research on a day-to-day basis, but also students who constitute our future scientific community.

### Bridging the gap: the role of pedagogical communities

Fostering a culture of open scholarship practices through communities (e.g., Framework for Open and Reproducible Research Training (FORRT; https://forrt.org), Collaborative Replications and Education Project (CREP; https://osf.io/wfc6u), ReproducibiliTea (https://reproducibilitea.org), Reproducibility for Everyone (R4E; https://www.repro4everyone.org), Open Science Communities (OSCs; https://www.openscience.nl), Principles and Practices of Open Research (PaPOR TraIL; https://osf.io/863ks), Teaching Integrity in Empirical Research (ProjectTier; https://www.projecttier.org), Reproducible Interpretable Open and Transparent Science Club (RIOT Science Club; http://riotscience.co.uk), Open Scholarship Knowledge Base (OSKB; https://www.oercommons.org/hubs/OSKB), and Berkeley Initiative for Transparency in the Social Sciences (BITSS; https://www.bitss.org) can bring important benefits to the academic community. Despite the different mission and scope of these initiatives, all are working towards integrating open scholarship into higher education while helping advance research integrity, transparency, reproducibility, and ethics through pedagogical reform. Pedagogical communities are key in facilitating the co-creation of open scholarship educational materials. Resources and didactics ‘*by educators for educators’* are crucial in facilitating the integration of open scholarship into higher education and reducing the burden placed on scholars. Pedagogical communities also offer a much-needed environment wherein scholars share individual experiences, identify common hurdles, and iteratively enhance their pedagogy towards better addressing the unique challenges ensuing from curricular reform. Through these exchanges, pedagogical communities help create a culture of open scholarship, benefiting those within the community, and those that interact with it.

Pedagogical communities also offer a low-entry point into improved research and pedagogical practices. As pedagogical communities welcome scholars from all levels, and often particularly early career researchers, they are an accessible space for educators wishing to learn and practice open scholarship. By cutting across career stages, these communities become essential to instilling the revised values and norms of open scholarship.

Further, pedagogical communities play a key role in offering a sense of community to those who would otherwise be deprived of such a learning opportunity when there are fewer top-down initiatives and infrastructure to encourage change. As such, these communities are essential to address recent concerns regarding the lack of diversity in the open scholarship movement, e.g., [17–21]. By breaking the boundaries of academic fields and geographical locations, such communities contribute to the advancement of social justice, making the movement more diverse and representative of the plural needs of academics.

We argue the integration of open scholarship into higher-education should not be seen as an additional layer to existing reform proposals—e.g., methodological reform, research ethics and integrity, societal impact, diversity and inclusion—but rather one that can unite them. Pedagogical communities provide an alternative to the current academic reality by creating and implementing fairer norms; building the foundations for an inclusive and safe environment welcoming to all people and perspectives; working towards crediting members for their work and helping them claim it; and creating didactic resources that unburden educators and unravel the hidden curricula. Whether focusing on creating and developing new methods of education, addressing the new challenges of curricular reforms ensuing from new and improved research norms, or highlighting the importance of epistemic, cultural, and demographic diversity, pedagogical communities are central to a broad range of solutions ensuing from the credibility revolution [5]. In sum, pedagogical communities go beyond educational and network purposes, working towards redefining the culture of open scholarship sustainably from within.

### A roadmap towards creating open pedagogies for open scholarship practices

Established in 2018, the Framework of Open and Reproducible Research Training (FORRT) is one such pedagogical community aiming to build, together with educators and students, a pathway to the stepwise adoption of principled, open teaching and mentoring practices, whilst also generating a conversation about the ethics and social impact of higher-education pedagogy. It responds to calls for a wider interpretation of open scholarship as inclusive scholarship, e.g., [21–23] by involving those at all stages of learning. In this sense, FORRT’s mission seeks to empower teachers and their students, who may find it otherwise challenging, to not only develop strong competencies in this area but also incorporate open scholarship into their teaching and learning.

To achieve its aims, FORRT has accomplished 12 unique initiatives to date [11], which also illustrate the role that pedagogical communities play in co-creating materials that lower barriers to entry into open scholarship (https://forrt.org/nexus). In a hackathon held at the 2021 Society for the Improvement of Psychological Science Annual Conference, the FORRT community drew from experts, interested parties, and stakeholders to co-create several evidence-based, publicly accessible lesson plans and > 60 ready-to-run activities that are accompanied by teaching notes and can be integrated into existing taught courses (see https://forrt.org/lesson-plans; [2]). This initiative addresses the lack of open source educational resources, which is essential to facilitate engagement with, and adherence to, research integrity and transparency, replicability, reproducibility, openness, and accessibility. Another important initiative aimed to deal with the overwhelming new (and ever-evolving) nomenclature in open scholarships, which can act as a barrier to incorporating open scholarship into higher education. Here, over 100 members of the FORRT community produced a consensus-based, editable Glossary of over 250 terms and their concise definitions with supporting references (https://forrt.org/glossary; [1]). The glossary provides a shared perspective and language to benefit researchers and teachers alike, whether experienced or newcomers to open scholarship, whilst also highlighting important considerations for social justice by making a wide range of accessibility and inclusivity-related terms well-represented within its language. Lastly, to reduce the burden on educators aiming to integrate open and reproducible practices into their teaching and mentoring, and aid in the learning process of any person interested in staying up-to-date with the open scholarship literature, FORRT has prepared over 200 summaries of academic articles related to varied topics on open and reproducible practices (https://forrt.org/summaries).

Taken together, these initiatives contribute to advance the open scholarship movement insofar as they provide scholars and educators with resources aiding the learning and subsequent integration of open principles into their research pipeline, teaching, and mentoring.

We hope to have exemplified how pedagogical communities bring important benefits to expand the reach of the open scholarship movement and create a culture of open scholarship involving scholars, educators, students, and consumers of science.

### Outlook

Although there is momentum behind improving research quality, longer-term and far-reaching change both in practice and in culture is only possible with initiatives that train high quality research practices within higher education. Regrettably, to date, the responsibility for incorporating open scholarship into education and training has heavily relied on the initiative of individual early adopters of the scholarship movement. Most initiatives lack support and financial incentives from academic institutions and thus governance, scholarly societies and funding agencies hold a vital role in the sustainability of such communities and subsequent impact.

The FORRT community has developed an Open Scholarship Glossary with more than 250+ defined terms, 200+ article summaries, lesson plans and + 60 activities. These are just three of twelve current FORRT initiatives, providing a rigorous and inclusive foundation for engaging with, and sharing, the open scholarship movement and yet these have been completed without funding and without substantive stakeholder investment. Science is a collaborative effort and we aim to better integrate with stakeholders in education and research to provide coherent support for these communities (e.g. in the form of recognition of open scholarship practices in hiring and promotion criteria, support for research knowledge exchange events, and facilitating cross-discipline collaboration to develop inclusive and widely applicable open scholarship teaching materials).

In conclusion, we (a) stress that it is critical to embed training in reproducibility and research integrity into higher education pedagogy to ensure long-term sustainable change; and (b) call for greater collaboration with pedagogical communities, paving the way for a much needed integration of top-down and grassroot open scholarship initiatives.

## Data Availability

All resources discussed in this article are available at forrt.org.

## Abbreviations

FORRT: Framework for Open and Reproducible Research Training
CREP: Crowdsourced Replication Project, ReproducibiliTea
R4E: Reproducibility for Everyone
OSCs: Open Science Communities
PaPOR TRaIL: Principles and Practices of Open Research: Teaching, Research, Impact, And Learning
ProjectTier: Teaching Integrity in Empirical Research
RIOT Science Club: Reproducible, Interpretable, Open, Transparent Science Club
OSKB: Open Scholarship Knowledge Base
BITSS: Berkeley Initiative for Transparency in the Social Sciences
